# Complete assignment of Ala, Ile, Leu^ProS^, Met and Val^ProS^ methyl groups of the protruding domain from human norovirus GII.4 Saga

**DOI:** 10.1007/s12104-020-09932-z

**Published:** 2020-01-28

**Authors:** Christoph Müller-Hermes, Robert Creutznacher, Alvaro Mallagaray

**Affiliations:** Center of Structural and Cell Biology in Medicine (CSCM), Institute of Chemistry and Metabolomics, University of Lübeck, Ratzeburger Allee 160, 23562 Lübeck, Germany

**Keywords:** Human norovirus, Protruding domain, NMR assignment, 4D HMQC-NOESY-HMQC, Site-specific isotope labelling, MILVA labelling

## Abstract

Attachment of human noroviruses to histo blood group antigens (HBGAs) is thought to be essential for infection, although how this binding event promotes infection is unknown. Recent studies have shown that 60% of all GII.4 epidemic strains may undergo a spontaneous post-translational modification (PTM) in an amino acid located adjacent to the binding pocket for HBGAs. This transformation proceeds with an estimated half-life of 1–2 days under physiological conditions, dramatically affecting HBGA recognition. The surface-exposed position of this PTM and its sequence conservation suggests a relevant role in immune escape and host-cell recognition. As a first step towards the understanding of the biological implications of this PTM at atomic resolution, we report the complete assignment of methyl resonances of a MIL^ProS^V^ProS^A methyl-labeled sample of a 72 kDa protruding domain from a GII.4 Saga human norovirus strain. Assignments were obtained from methyl–methyl NOESY experiments combined with site-directed mutagenesis and automated assignment. This data provides the basis for a detailed characterization of the PTM-driven modulation of immune recognition in human norovirus on a molecular level.

**Electronic supplementary material:**

The online version of this article (10.1007/s12104-020-09932-z) contains supplementary material, which is available to authorized users.

## Biological context

Noroviruses are the leading cause of viral gastroenteritis in humans. (Ahmed et al. 2014; de Graaf et al. 2016) While not often a fatal disease in the developed world, noroviruses are responsible for ~ 4.2 billion dollars in direct healthcare costs per year worldwide. (Bartsch et al. 2016) Efforts to develop broadly active vaccines have been thwarted by the ability of noroviruses to constantly evolve, generating new strains every 2–4 years. (de Graaf et al. 2016; Mallory et al. 2019; Melhem 2016; van Beek et al. 2018) Human noroviruses (hNoV) require attachment to histo blood group antigens (HBGAs) as first step for infection (Baldridge et al. 2016; Nasir et al. 2017; Taube et al. 2018). There is compelling evidence that the structure and composition of the binding pocket for HBGA recognition is highly conserved among the prevalent norovirus strains (Singh et al. 2015a, b) and therefore, it has been targeted for the development of entry inhibitors (Heggelund et al. 2017; Koromyslova et al. 2017; Koromyslova et al. 2015; Morozov et al. 2018; Netzler et al. 2019; Prasad et al. 2016; Shang et al. 2013). However, the lack of effective human norovirus cell culture systems at the time these studies were conducted precluded their development beyond the stage of hit discovery (Oka et al. 2018). Recently, two cell culture systems and an animal model for human noroviruses have been established, opening the possibility for infection assays in the near future (Ettayebi et al. 2016; Jones et al. 2015; Van Dycke et al. 2019; Zhang et al. 2013). Interestingly, it turned out that besides HBGAs, bile acids are important co-factors required to promote norovirus infection (Bartnicki et al. 2017; Ettayebi et al. 2016). Although the underlying mechanisms are not yet well understood, the structural basis for murine norovirus (Nelson et al. 2018) and human norovirus (Creutznacher et al. 2019; Kilic et al. 2019) binding to bile acids have already been addressed.

The viral capsid of human noroviruses consists of 180 copies of a single capsid protein VP1. The highly conserved HBGA binding pocket is located in the outer-most region of the protruding domain (P-domain) of the VP1 protein. Expression of protruding domains in *Escherichia coli* affords the so-called P-dimers, a 72 kDa homodimer that retains the specificity towards HBGAs and bile acids. Recently, we have obtained an almost complete backbone assignment of the P-domain of a human epidemic strain, GII.4 Saga4/2006 (Mallagaray et al. 2019). Unexpectedly, the NMR assignment exposed a fast and highly specific deamidation of Asn373, resulting in an isopeptide linkage and abrogating HBGA recognition. Chemical shift perturbation (CSP)-based titrations using l-fucose and b-trisaccharide disclosed dissociation constants *K*_D_ in the mM range. CSPs were observed as far as 25 Å from the HBGA binding pocket at ligand saturation, suggesting the presence of an allosteric network regulated by HBGA binding. A recent study monitored the binding of human norovirus virus-like particles to glycosphingolipids embedded in a phospholipid bilayer (Parveen et al. 2019). From competitive titrations, it was concluded that the intrinsic (per binding site) bond energies of H type 1 and B type 1 glycosphingolipids are in good agreement with the *K*_D_s reported for HBGAs by NMR. It seems that affinities of hNoV for HBGAs had been significatively overestimated in previous studies. In addition, the impact of deamidation in early publications remains unclear, since at the time these studies were conducted the post-translational modification in hNoV had not been discovered.

Methyl TROSY experiments yield high-quality spectra even for protein ensembles far over 100 kDa (Schütz and Sprangers 2019; Tugarinov et al. 2003). The increased sensitivity obtained by means of transverse relaxation optimized spectroscopy (TROSY) (Riek et al. 1999) combined with selective isotopic methyl-labeling in a perdeuterated background allows for fast spectral acquisition and high resolution even for samples containing low protein concentrations (Gossert and Jahnke 2016). This becomes critical for costly and unstable proteins, as is the case for Saga P-dimers. Hence, the assignment of the MIL^ProS^V^ProS^A methyl-labeled P-dimers is essential for a precise biophysical characterization of HBGA and antibody recognition by noroviruses. These results will pave the way for the design of potent and selective entry inhibitors against human norovirus.

## Methods and experiments

### Protein expression and purification

Non-deamidated [*U*-^2^H] ^13^C-methyl labeled GII.4 Saga4/2006 P domains (GenBank accession number AB447457, residues 225 to 530) were expressed according to a modified version of previously described protocols (Creutznacher et al. 2019; Mallagaray et al. 2019). Mutants I293L, I317L, V413A and L507V from GII.4 Saga4/2006 protruding domain were generated by site-directed mutagenesis (Eurofins Genomics). Mutations were confirmed by DNA sequencing. Primers used for mutagenesis are listed int Table 1.

**Table 1:** List of primers used for site-directed mutagenesis

Entry	Mutant	Primers
1	I293L	Forward: 5′-CGCGGTGATGTTACCCATCTGGCCGGTAGCCGTAATTATAC-3′ Reverse: 5′-GTATAATTACGGCTACCGGCCAGATGGGTAACATCACCGCG-3′
2	I317L	Forward: 5′-GGAATAATTATGACCCGACCGAAGAACTGCCGGCACCGCTGGG-3′ Reverse: 5′-CCCAGCGGTGCCGGCAGTTCTTCGGTCGGGTCATAATTATTCC-3′
3	V413A	Forward: 5′-CTATAGCGGTCGTAATGCGCATAATGTGCATCTGGCACCG-3′ Reverse: 5′-CGGTGCCAGATGCACATTATGCGCATTACGACCGCTATAG-3′
4	L507V	Forward: 5′-GGTCAGCATGATGTGGTTATTCCGCCTAATGGC-3′ Reverse: 5′-GCCATTAGGCGGAATAACCACATCATGCTGACC-3′

For protein expression, *E. coli* BL21(DE3) were transformed with a pMal-c2x expression vector encoding the genes for ampicillin resistance, a fusion protein of maltose-binding protein (MBP), two His-tags, a HRV3C cleavage domain and the P-domain. Due to the cloning strategy, P-domains contain an extra GPGS sequence preceding K225. Bacteria were grown in 25 ml of supplemented TB medium at 37 °C for 6–8 h. A volume containing cells enough to give an OD_600_ of 0.1 in a 20 ml culture was spun down (11000xg at room temperature), the supernatant was discarded and the pellet was resuspended in 20 ml of M9^+^/D_2_O minimal medium. The culture was incubated overnight at 37 °C. Like before, a culture volume for a final OD_600_ of 0.1 in 20 ml was spun down, the supernatant was discarded, the pellet resuspended in 40 ml of M9^+^/D_2_O minimal medium and incubated at 37 °C until an OD_600_ of 0.4–0.5. The culture was diluted to the final volume (normally 250 ml) by addition of M9^+^/D_2_O, and cells were incubated at 37 °C until an OD_600_ of 0.6–0.8 was reached. 20 ml of the solution containing the isotopically labeled precursors were added and the culture was incubated at 16 °C for 1 h. Protein overexpression was induced with 1 mM isopropylthiogalactoside (IPTG). Growth was continued at 16 °C until stationary phase was reached (normally after 4–5 days). To maintain the antibiotic pressure, 100 µg/ml ampicillin were added every 36 h. Cells were harvested by centrifugation at 5000×*g* for 20 min at 4 °C, and pellets were stored at − 80 °C. For details on the preparation of supplemented TB medium, M9^+^/D_2_O minimal medium and isotopically labeled precursors see supplementary material.

For protein purification, the pellet was resuspended in PBS buffer and then lysed using a high pressure homogenizer (Thermo). The lysate was clarified by centrifugation, and the fusion protein was purified using a Ni-NTA resin (Qiagen). MBP and the His-tag were cleaved from the P-domain using HRV 3C protease (Novagen). Cleaved P-domain protein eluted from Ni-NTA resin and was further purified by size-exclusion chromatography using a Superdex 26/600 75 pg column (GE Healthcare) in 20 mM sodium phosphate buffer (pH 7.3). Protein purity and dimer concentration were monitored by SDS-polyacrylamide electrophoresis and UV absorption (ε280 70,820/M/cm), respectively. Separation of native and deamidated P-dimer species was achieved by cation exchange chromatography using a 6 ml Resource S column (GE Healthcare). Protein samples were prepared in 20 mM sodium acetate buffer (pH 4.9) and eluted using a 78.4 ml linear salt gradient up to 195 mM NaCl with a flowrate of 1 ml per min. All steps were conducted at 6 °C. Samples were kept in this buffer at 6 °C to slow the deamidation reaction.

### Sample preparation and NMR spectroscopy

Saga P-dimers were exchanged into NMR buffer using Zeba™ Spin Desalting Columns (MWCO 40 KDa, Thermo Scientific), which had been pre-equilibrated in the NMR buffer. The abundance of non-deamidated Saga P-dimers during NMR experiments was monitored from the ^1^H,^13^C HMQC spectra. NMR samples were prepared in 3 mm NMR tubes in 75 mM sodium phosphate, pH* 7.40, 100 mM NaCl, 100 µM DSS-d_6_, 100 µM imidazole and 0.02% NaN_3_ in > 99.9% D_2_O. We used the imidazole signals as internal standard to monitor possible pH drifts during the measurements produced by NH_3_ released due to deamidation of P-dimers. (Baryshnikova et al. 2008)

For identification of residue types, 2D ^1^H,^13^C HMQC experiments (Tugarinov et al. 2003) were conducted with P-dimer concentrations ranging between 25–200 µM with 1024 × 512 points in the direct and indirect dimensions, respectively. Spectra were acquired for [*U*-^2^H], δ1-[^13^C^1^H_3_]-Ile-labeled (I), [*U*-^2^H], δ1-[^13^C^1^H_3_]-Ile, γ1,2-[^13^C^1^H_3_]-Val-labeled (IV), [*U*-^2^H], δ1-[^13^C^1^H_3_]-Ile, γ2-[^13^C^1^H_3_]-Val, δ2-[^13^C,^1^H_3_]-Leu-labeled (IL^ProS^V^ProS^), [*U*-^2^H], ε-[^13^C,^1^H_3_]-Met, δ1-[^13^C^1^H_3_]-Ile, γ2-[^13^C^1^H_3_]-Val, δ2-[^13^C,^1^H_3_]-Leu-labeled (MIL^ProS^V^ProS^) and [*U*-^2^H], ε-[^13^C,^1^H_3_]-Met, δ1-[^13^C^1^H_3_]-Ile, γ2-[^13^C^1^H_3_]-Val, δ2-[^13^C,^1^H_3_]-Leu, β-[^13^C^1^H_3_]-Ala-labeled (MIL^ProS^V^ProS^A) P-dimers.

2D HMQC-NOESY spectra were acquired using a standard Bruker pulse sequence (hmqcetgpno) with 50, 100, 150, 200 and 300 ms mixing times (*t*_mix_) and 1024 × 400 data points in the direct and indirect dimensions, respectively. Intensities for ten randomly selected NOE cross-peaks were extracted and plotted against *t*_mix_. Based on the NOE build-up curves, a *t*_mix_ of 125 ms was selected for the 4D HMQC-NOESY-HMQC experiment. The 4D HMQC-NOESY-HMQC experiment (Tugarinov and Kay 2004; Tugarinov et al. 2005; Wen et al. 2012) was recorded using 30% non-uniform sampling (NUS) according to a Poisson Gap sampling schedule (Hyberts et al. 2010) with 9329 complex NUS data points in a grid of 52 (^13^C) × 92 (^1^H) × 52 (^13^C) points in the indirect dimensions. 1024 were acquired in the direct dimension. The spectrum was processed on a Mac-BookPro running Yosemite 10.10.5 using Multi-Dimensional Decomposition (MDD, Bruker).

All NMR spectra were recorded on a Bruker AV III 500 MHz NMR spectrometer equipped with a TCI cryogenic probe at 298 K. If not stated otherwise, spectra were processed with TopSpin 4.0.2 and peak positions and intensities were extracted using CCPNMR Analysis 2.4.2 software suit (Vranken et al. 2005). ^1^H chemical shifts were referenced according to the DSS-d_6_ peak, and ^13^C signals were referenced indirectly. A complete list of samples prepared and experiments performed can be found in Table 2.

**Table 2:** List of samples prepared in this study

Entry	Labeling	Construct	Concentration (µM)	NMR experiment
1	MIL^ProS^V^ProS^A	Wt	200	4D HMQC-NOESY-HMQC, 2D HMQC-NOESY, ^1^H,^13^C HMQC
2	MIL^ProS^V^ProS^	Wt	75	^1^H,^13^C HMQC
3	IL^ProS^V^ProS^	Wt	60	^1^H,^13^C HMQC
4	IV	Wt	115	^1^H,^13^C HMQC
5	I	Wt	50	^1^H,^13^C HMQC
6	IL^ProS^V^ProS^	I293L	55	^1^H,^13^C HMQC
7	IL^ProS^V^ProS^	I317L	37	^1^H,^13^C HMQC
8	IL^ProS^V^ProS^	V413A	55	^1^H,^13^C HMQC
9	IL^ProS^V^ProS^	L507V	41	^1^H,^13^C HMQC

### Assignment and data deposition

Several strategies have been developed for the assignment of protein methyl groups in the recent years. Classic approaches rely on systematic site-directed mutagenesis (Amero et al. 2011). However, this approach demands the preparation of many isotopically labeled samples, and can be very cost-intensive. Pulse sequences transferring magnetization from backbone amides or carbonyls to side chain methyl groups can be used when backbone assignments are available (Tugarinov and Kay 2003). Recently, structure-based assignment strategies have emerged as a powerful alternative, allowing the assignment of supramolecular structures of hundreds of kDa (Proudfoot et al. 2016; Sprangers and Kay 2007; Velyvis et al. 2009; Xiao et al. 2015). Such approaches require high-resolution crystal structures of the protein of interest, which serve as a structural scaffold for the assignment. Short- and long-range spatial restraints can be obtained from 3D or 4D HMQC-NOESY experiments (Tugarinov et al. 2005; Wen et al. 2012) and paramagnetic NMR experiments (Flugge and Peters 2018; Venditti et al. 2011), respectively. Several algorithms have been developed to perform structure based methyl group assignments automatically, as is the case for *MAP-XSII* (Xu and Matthews 2013), *FLAMEnGO* (Chao et al. 2014), *MAGMA* (Pritisanac et al. 2017) and *MAGIC* (Monneau et al. 2017). Spatial restraints can be combined with chemical shift predictions to increase number of assignments (Han et al. 2011; Sahakyan et al. 2011).

**Fig. 1:**
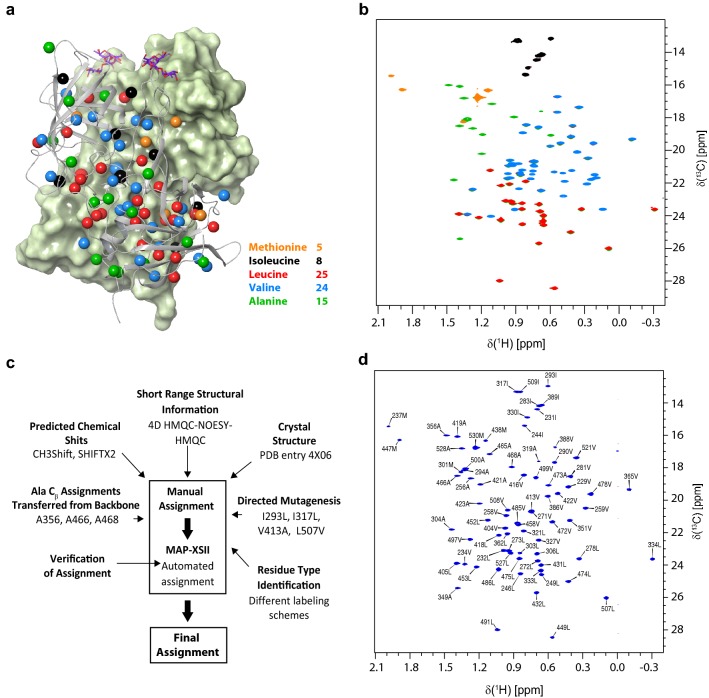
**a** Crystal structure of Saga P-dimers (PDB entry 4X06). One monomer is depicted as a grey cartoon and the other as a pale green molecular surface. For clarity, only the *pro-S* methyl groups of Leu and Val are shown. A summary of the amino acids labeled is provided in the middle. B-trisaccharide is shown in purple sticks only as orientation, but was not added to the samples. **b** Superposition of ^1^H,^13^C HMQC spectra. Each spectrum is colored individually (I black, IV blue, IL^ProS^V^ProS^ red, MIL^ProS^V^ProS^ orange and MIL^ProS^V^ProS^A green). Carbons of ^13^C-labeled methyl groups in one monomer are highlighted as spheres and colored with the same color scheme as in **a**. **c** Strategy followed for the assignment of the Ala, Ile, Leu^ProS^, Met and Val^ProS^ methyl groups from GII.4 Saga P-dimers. **d**^1^H,^13^C HMQC spectrum acquired for a MIL^ProS^V^ProS^A sample showing the complete assignment. All samples were prepared in Na phosphate 75 mM and NaCl 0.1 M pH* 7.40 except the I labeled sample, which was prepared in Tris 25 mM and NaCl 0.3 M pH* 7.25. All samples were measured at 500 MHz and 298 K.

Here, we used a structure-based strategy to obtain the sequence-specific assignments of the methyl groups of MIL^ProS^V^ProS^A ^13^C-methyl labeled P-domains from GII.4 Saga4/2006. Methyl groups from Met, Ile δ1, Leu δ2 (*pro-S*), Val γ2 (*pro-S*) and Ala β were ^13^C labeled in a perdeuterated background, yielding a total of 77 ^13^C-methyl groups to be assigned. The labeling pattern was chosen to maximize the methyl probe density and to minimize signal overlapping. The use of stereoselective labeling with Val^ProS^ and Leu^ProS^ employing 2-(^13^C)methyl-4-(^2^H_3_)-acetolactate (Gans et al. 2010; Tugarinov et al. 2003) as precursor greatly reduced signal overlapping in the usually crowded Leu and Val spectral region. Methyl groups are well distributed over the entire P-domain, as can be seen from Fig. 1a and S1. In a first step, the methyl resonances were assigned to their respective amino acid types. Five differently ^13^C-methyl labeled Saga P-dimers samples were prepared for the amino acid type differential assignment. Specifically, we synthesized I-, IV-, IL^ProS^V^ProS^-, MIL^ProS^V^ProS^- and MIL^ProS^V^ProS^A-methyl labeled P-dimers. (Fig. 1b and Table 2, entries 1 to 5). Comparison of the 2D ^1^H,^13^C HMQC spectra delivers unambiguous assignment of each cross peak to its corresponding amino acid type.

The 4D methyl–methyl NOESY experiment provided 174 unambiguous cross-peaks, which were the only spatial restraints during the assignment process. A scheme showing the complete NOE network can be inspected in the supplementary material (Fig. S2). As structural model, the crystal structure with PDB code 4 X06 was selected because of its high resolution. In a first attempt, 69 out of 77 peaks could be assigned following the rational described in (Xiao et al. 2015). The assignment of amino acids I293, I317, V413 and L507 by site-directed mutagenesis combined with the transfer of C_β_ assignments from a previously published backbone assignment (Mallagaray et al. 2019) for A356, A466 and A468 resulted in a 100% assignment of the MIL^ProS^V^ProS^A-methyl labeled P-dimers.

**Fig. 2:**
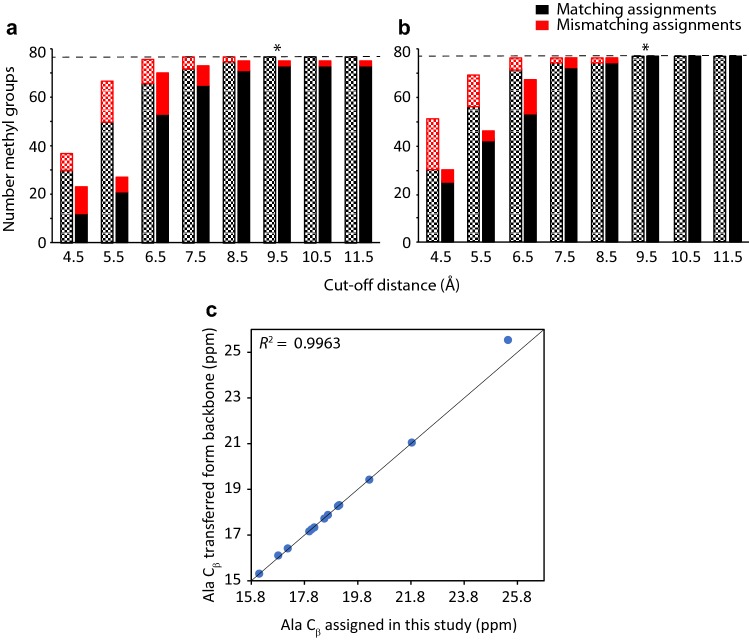
Results of *MAP-XSII* MMC tests for **a** different cut-off distances exclusively using 4D HMQC-NOESY-HMQC data, and **b** after the addition of seven independently assigned amino acids. *MAP-XSII* requires that the program is run at a number of distance thresholds, and the distance that provides the most confident assignments is taken as the final value, here indicated with an asterisk. Dotted bars correspond to *MAP-XSII* runs using predicted chemical shifts from *CH3Shift* and *SHIFTX2*, whilst no chemical shift prediction was used for the filled bars. Black indicates number of matches with the manual assignment, and red mismatches. For all the tests, protocols were executed as described in the original paper. **c** Correlation between assignments for Ala C_β_ obtained in this study and the ones transferred from a previous assignment (BMRB accession number 27445). A correcting factor of 0.78 was included to compensate for the deuterium isotope effect (see Table S2).

For validation, we computed an independent assignment based on *MAP-XSII* (Xu and Matthews 2013). *MAP-XSII* performs a fully automated assignment based on experimental restraints and a structural model supplied. Chemical shifts of Ala, Ile, Leu (*pro-S*) and Val (*pro-S*) were predicted using *CH3Shift* (Sahakyan et al. 2011), and Met chemical shifts were calculated with *SHIFTX2* (Han et al. 2011). Twenty Metropolis Monte Carlo (MMC) tests were computed for cut-off distances between 4.5 and 11.5 Å, and the ten runs showing the highest scores at each cut-off distance were selected for assignment. Calculations were run with and without using the seven independent assignments as restraints. Automated and manual assignments were compared, and results were plotted in Fig. 2a, b (dotted bars). In both scenarios, a complete match between manual and automated assignments was observed over 8.5 Å threshold distances. However, it is worth mentioning that the inclusion of predicted chemical shift in the automated assignment increases the number of assignments, although at a significant cost of accuracy (Pritisanac et al. 2017). Thus, when *MAP-XSII* was run without predicted chemical shifts a 100% match between manual and automated assignments was again obtained over 8.5 Å with the inclusion of the seven independent assignments, thus validating our preliminary manual assignment (Fig. 2a, b, filled bars). Finally, we used the Ala C_β_ assignments as semi-quantitative indicator of the goodness of the assignment. Alanines can be considered as a good reporter of the quality of the assignment, since they are present in almost every NOE cluster. Ala C_β_ chemical shifts obtained in this study matched perfectly with a previously reported backbone assignment (Mallagaray et al. 2019) after the corresponding correction for the deuterium isotope effect (Fig. 2c).

The chemical shift assignments for non-deamidated MIL^ProS^V^ProS^A ^13^C-methyl labeled P-domains from GII.4 Saga4/2006 have been deposited in the BioMagResBank (http://www.bmrb.wisc.edu) under the accession number 28030. A list of chemical shifts of amino acids showing chemical shifts perturbations > 1 σ after conversion of Asn373 into isoD373 can be found in the supplementary material (Table S3).

## Electronic supplementary material

Below is the link to the electronic supplementary material.

Electronic supplementary material 1 Information about protein expression and purification, details on the NOE network, comparison of chemical shifts for Ala C_β_ and chemical shifts for the deamitaed form of GII.4 Saga4/2006 P-dimers can be found in the Online Resource 1. (DOCX 562 kb)
