# Supplementary material for: Complete assignment of Ala, Ile, LeuProS, Met and ValProS methyl groups of the protruding domain from human norovirus GII.4 Saga
Source: Biomol NMR Assign. 2020 Jan 28;14(1):123–30. doi: 10.1007/s12104-020-09932-z (PMC7069894; doi:10.1007/s12104-020-09932-z)
Supplement: Supplementary file 1 — Electronic supplementary material 1 Information about protein expression and purification, details on the NOE network, comparison of chemical shifts for Ala Cβ and chemical shifts for the deamitaed form of GII.4 Saga4/2006 P-dimers can be found in the Online Resource 1. (DOCX 562 kb) [file 12104_2020_9932_MOESM1_ESM.docx]

**Supplementary material – Biomolecular NMR Assignments**

**Complete assignment of Ala, Ile, Leu^ProS^, Met and Val^ProS^ methyl groups of the protruding domain from human Norovirus GII.4 Saga**

Christoph Müller-Hermes, Robert Creutznacher and Alvaro Mallagaray^*^

University of Luebeck, Center of Structural and Cell Biology in Medicine (CSCM), Institute of Chemistry and Metabolomics, Ratzeburger Allee 160, 23562 Luebeck, Germany

**Table of Contents**

1. Protein expression and purification 3
2. Distribution of amino acids containing ^13^C-labeled methyl groups in the sequence 4
3. Schematic representation of NOE network obtained from 4D HMQC-NOESY-HMQC experiments 4
4. Comparison of chemical shifts obtained for Ala C_β_ from the structure-based and the backbone assignments 5
5. Chemical shifts of amino acids showing chemical shifts perturbations > 1 σ after conversion of Asn373 into isoD373 5

**Protein expression and purification of [*U*-^2^H] ^13^C-methyl labeled P-dimers**

*Supplemented Terrific Broth medium (for 1 L):* 12 g tryptone, 24 g yeast extract and 40 ml glycerol per liter culture supplemented with M9 minimal medium components (0.5 g of NaCl, 3.3 g of KH_2_PO_4_, 16.6 g of Na_2_HPO_4_x12H_2_O, 1 g of NH_4_Cl, 1 ml of 1 M MgSO_4_, 1 ml of 0.1 M CaCl_2_ and 0.2 % glucose per liter culture), 0.4% casamino acids and 100 µg/ml ampicillin.

*M9^+^/D_2_O minimal medium (for 250 mL):* Mix 3.25 g Na_2_HPO_4_*2H_2_O, 0.9 g anhydrous KH_2_PO_4_, 0.25 g NaCl, 0.75 g deuterated D-glucose (1,2,3,4,5,6,6-d_7_) and 0.75 g NH_4_Cl in 20 ml D_2_O and lyophilize. Prepare another solution containing 116.25 mg of MgSO_4_, 50 mg of MgCl_2_, 3.55 mg of CaCl_2_ and 5 mg of vitamin B1 in 5 ml D_2_O and lyophilize. Prepare the 100x vitamins solution in 10 ml D_2_O, which contains 0.1 mg of riboflavin and 1 mg of each of the following compounds: D-biotin, choline chloride, folic acid, nicotinamide, D-pantothenic acid, pyridoxal hydrochloride and cobalamine, all obtained from Aldrich. Dissolve the lyophilized powders and 2.5 ml of the 100x vitamins solution in 250 ml D_2_O. Adjust pH* to 7.50, add 100 µg/ml ampicillin and filter under sterile conditions. The medium should be used within the following 24 h after preparation.

*Solution containing the isotopically labeled precursors:* For 250 ml of M9^+^/D_2_O minimal medium, only 20 ml are required. Mix 260 mg Na_2_HPO_4_*2H_2_O, 72 mg anhydrous KH_2_PO_4_, 20 mg NaCl, 60 mg deuterated D-glucose (1,2,3,4,5,6,6-d_7_), 60 mg NH_4_Cl, 9.3 mg of MgSO_4_, 4 mg of MgCl_2_, 0.28 mg of CaCl_2_ and 0.4 mg of vitamin B1 in 5 ml D_2_O and lyophilize. Mix the lyophilized powders, the desired labeled amino acids (see Table S1) and 20 µl of the 100x vitamins solution and add D_2_O up to 20 ml. Adjust pH* to 7.50, add 100 µg/ml ampicillin and filter under sterile conditions. The medium should be used within the following 24 h after preparation.

*Preparation of 2-keto-3,3-D_2_-4-^13^C-butyric acid:* 60 mg of 2-keto-4-^13^C-butyric acid were dissolved in 50 mL D_2_O containing 142 mg anhydrous KH_2_PO_4_ and 240 mg of anhydrous Na_2_HPO_4_. The pH* was adjusted to 10.5 with NaOD, and the solution was stirred overnight at 45 °C. The H/D exchange was verified by NMR spectroscopy, and the pH* was adjusted to 7.4 with DCl. The solution was sterile filtered into 5 mL aliquots (6 mg 2-keto-3,3-D_2_-4-^13^C-butyric acid each) and stored at -20 °C.

**Table S1:** Precursor used for the different ^13^C-methyl labeling schemes.

| **Labeled amino acid** | **Precursor** | **Quantity per 100 ml culture** |
| --- | --- | --- |
| Ile | 2-keto-3,3-D_2_-4-^13^C-butyric acid^1^ | Aliquot á 6 mg |
| Leu^proS^, Val^proS^ | 2-(^13^C)methyl-4-(^2^H_3_)-acetolactate^2^ | Aliquot á 20 mg |
| Val | 3-methyl-^13^C-3,4,4,4-D_4_−α-ketoisovaleric acid, sodium salt^3^  *E. coli* OD2 D medium^4^ | 38 mg  24 ml |
| Met | L-Methionine-(6-^13^C)^5^ | 20 mg |
| Ala | L-alanine-(3-^13^C, 2-D)^6^ | 40 mg |
|  | Succinate-D_4_^7^ | 250 mg |

^1^Prepared from 2-keto-4-^13^C-butyric acid (CortecNet) as described above. Precursors were obtained from ^2^NMR-Bio, ^3,6^Eurisotop, ^4^Silantes and ^5,7^Aldrich. ^4^Was directly added to the M9^+^/D_2_O minimal medium.


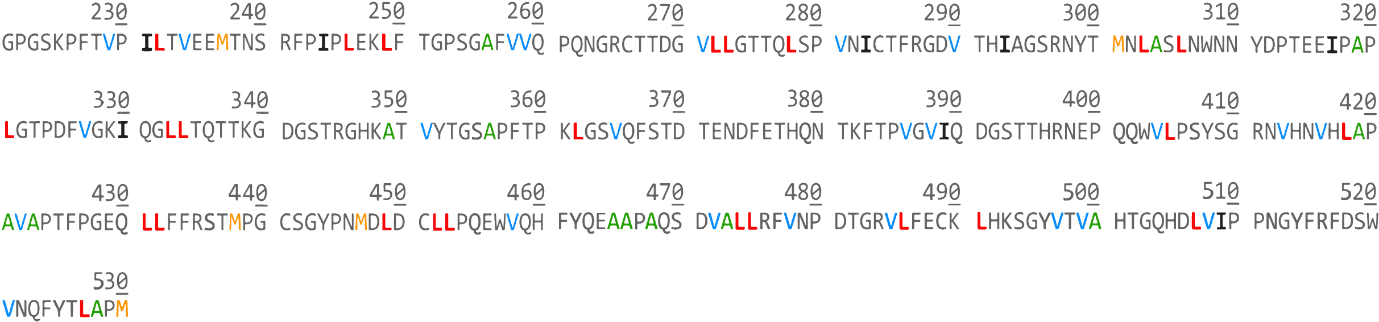


**Figure S1**. Distribution of amino acids containing ^13^C-labeled methyl groups depicted in the sequence of GII.4 Saga protruding domain. The numbering relates to the VP1 protein, which includes the shell domain. ^13^C-methyl labeled amino acids have been color coded according to Figure 1.


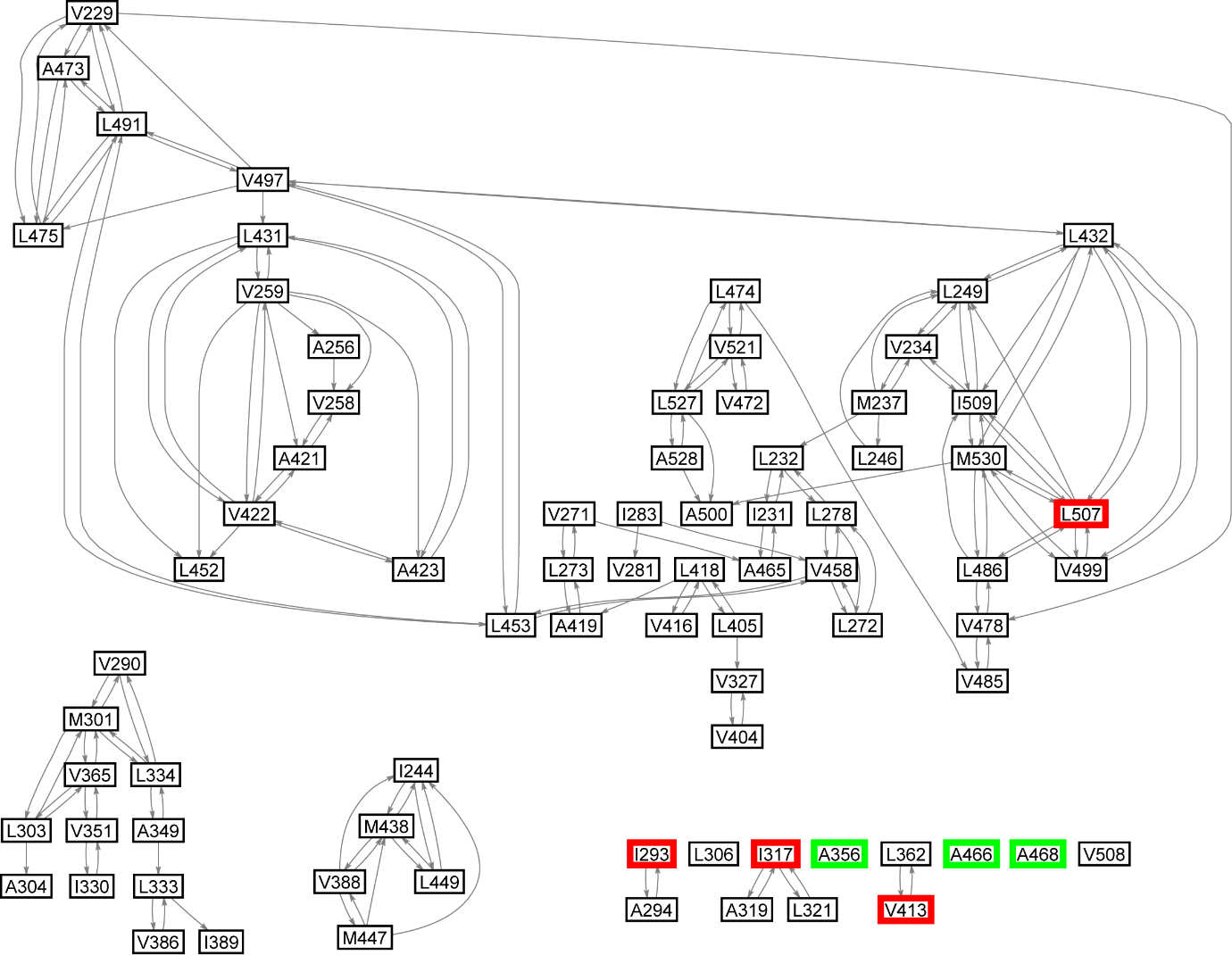


**Figure S2.** Schematic representation of the NOE network obtained from the 4D HMQC-NOESY-HMQC experiment with MIL^ProS^V^ProS^A ^13^C-methyl labeled GII.4 Saga P-dimers. Arrow origins indicate the peak for which F1 (^1^H) and F2 (^13^C) frequencies have been selected in the 4D HMQC-NOESY-HMQC. Arrow heads denote NOEs observed in the F3–F4 plane for that particular methyl group. Amino acids assigned by directed mutagenesis are indicated in red, and assignments transferred from the backbone are indicated in green. V508 and L306 were assigned by exclusion. Their assignments were validated by chemical shift prediction, and in the case of V508 by the chemical shift perturbations introduced by L507V.

**Table S2:** Comparison of chemical shifts obtained for Ala C_β_ from the structure-based (BMRB accession number 28030) and the backbone assignments (BMRB accession number 27445).

| **N°** | **Amino acid number** | **Chem. Shift for C_β_ structure-based assignment (ppm)** | **Chem. Shift for C_β_ backbone assignment (ppm)** | **Chem. Shift difference (ppm)** |
| --- | --- | --- | --- | --- |
| 1 | 256 | 18.661 | 17.883 | 0.778 |
| 2 | 294 | 18.146 | 17.335 | 0.811 |
| 3 | 304 | 21.808 | 21.051 | 0.757 |
| 4 | 319 | 17.606 | - | - |
| 5 | 349 | 25.421 | 25.540 | 0.881 |
| 6 | 356 | 16.003 | - | - |
| 7 | 419 | 16.078 | 15.308 | 0.770 |
| 8 | 421 | 19.035 | 18.270 | 0.765 |
| 9 | 423 | 20.214 | 19.429 | 0.785 |
| 10 | 465 | 17.149 | 16.416 | 0.733 |
| 11 | 466 | 18.528 | 17.727 | 0.801 |
| 12 | 468 | 17.955 | 17.162 | 0.793 |
| 13 | 473 | 19.084 | 18.313 | 0.771 |
| 14 | 500 | 18.046 | 17.250 | 0.796 |
| 15 | 528 | 16.792 | 16.112 | 0.680 |

**Table S3:** ^1^H and ^13^C chemical shifts of MIL^ProS^V^ProS^A labeled isoD373 Saga P dimers. Only methyl groups showing chemical shift perturbations (CSPs) over 1 σ are listed. Leu *pro-S* corresponds to Leu *delta2*, and Val *pro-S* corresponds to Val *gamma2*.

| **Sequence**  **position** | **Residue**  **type** | **Methyl**  **position** | **^1^H (ppm)** | **^13^C (ppm)** |
| --- | --- | --- | --- | --- |
| 290 | Val | gamma2 | 0.54 | 17.66 |
| 293 | Ile | delta1 | 0.60 | 12.96 |
| 294 | Ala | beta | 1.33 | 18.15 |
| 301 | Met | epsilon | 1.36 | 18.27 |
| 333 | Leu | delta2 | 0.66 | 24.33 |
| 334 | Leu | delta2 | -0.31 | 23.62 |
| 349 | Ala | beta | 1.39 | 25.42 |
| 351 | Val | gamma2 | 0.41 | 21.24 |
| 365 | Val | gamma2 | -0.10 | 19.34 |

CSPs were calculated as Euclidean distances Δν_Eucl_ in Hz according to Eq. 1: (Shi and Kay 2014)

$\Delta v_{Eucl} \left( Hz \right)=\sqrt{{\Delta v_{H}}^{2}+{\Delta v_{C}}^{2}}$ Eq. 1

With Δν_H_ and Δν_C_ being the CSPs of ^1^H and ^13^C resonances, respectively, observed between the wild type (Asn373) and the deamidated (isoD373) Saga P-dimers.

**References:**

Shi L, Kay LE (2014) Tracing an allosteric pathway regulating the activity of the HslV protease Proc Natl Acad Sci U S A 111:2140-2145 doi:10.1073/pnas.1318476111
